# The prognostic potential of alkaline phosphatase and lactic acid dehydrogenase in bmCRPC patients without significant PSA response under enzalutamide

**DOI:** 10.1186/s12885-022-09483-7

**Published:** 2022-04-08

**Authors:** Renata Poteska, Kambiz Rahbar, Axel Semjonow, Andres Jan Schrader, Martin Boegemann, Katrin Schlack

**Affiliations:** 1 Department of Urology, University Hospital Muenster, Münster, Germany; 2 Department of Nuclear Medicine, University Hospital Muenster, Münster, Germany; 3 University Hospital Muenster, Westdeutsches Tumorzentrum, Münster, Germany; 4 Department of Urology, University Hospital Muenster, Albert-Schweitzer-Campus 1, GB A1, D-48149 Muenster, Germany

**Keywords:** bmCRPC, Enzalutamide, Prognosis, Prostate specific antigen, Alkaline phosphatase, Lactate dehydrogenase

## Abstract

**Background:**

In patients with bone metastatic castration-resistant prostate cancer (bmCRPC) on systemic treatment, it is difficult to differentiate between continuous rise of prostate specific antigen (PSA) representing progression, and PSA-surge, which is followed by clinical response or stable disease. The purpose of this study was to evaluate the prognostic value of dynamic changes of alkaline phosphatase (ALP) and lactic acid dehydrogenase (LDH) levels as a predictor of clinical efficacy or therapeutic resistance of patients who do not show a sufficient initial PSA decline of ≥50% from baseline during early therapy with Enzalutamide.

**Methods:**

Forty-eight men with bmCRPC on Enzalutamide 07/2010-09/2019 with initially rising PSA were analyzed. We monitored PSA, LDH and ALP at week 0, 2, 4, and every 4 weeks thereafter and analyzed the correlation between ALP rising at 12 weeks with or without LDH-normalization and the association with survival. For this we used Kaplan Meier analysis and uni- and multivariate cox-regression models.

**Results:**

In Kaplan-Meier analysis, ALP rising at 12 weeks with or without LDH-normalization was associated with significantly worse median progression-free survival (PFS) of 3 months vs. 5 months (Log rank *P* = 0.02) and 3 months vs. 5 months (*P* = 0.01), respectively and overall survival (OS) with 8 months vs. 15 months (*P* = 0.02) and 8 months vs. 17 months (*P* < 0.01). In univariate analysis of PFS, ALP rising at 12 weeks alone, ALP rising at 12 weeks without LDH-normalization and application of Enzalutamide after chemotherapy showed a statistically significant association towards shorter PFS (hazard ratio (HR): 0.51,* P* = 0.04; HR: 0.48, *P* = 0.03; HR: 0.48, *P* = 0.03). Worse OS was significantly associated with ALP rising at 12 weeks alone, ALP rising at 12 weeks without LDH-normalization, and application of Enzalutamide after chemotherapy (HR: 0.47, *P* = 0.02; HR: 0.36, *P* < 0.01; HR: 0.31, *P* < 0.01). In multivariate analysis only the application of Enzalutamide after chemotherapy remained an independent prognostic factor for worse OS (HR: 0.36, *P* = 0.01).

**Conclusions:**

Dynamic changes of ALP (non-rise) and LDH (normalization) under therapy with Enzalutamide may be associated with clinical benefit, better PFS, and OS in patients with bmCRPC who do not show a PSA decline.

## Background

Prostate cancer (PCa) is the most frequent type of cancer and the second most common reason of cancer-related deaths in men [1].

Mostly, metastatic disease develops from locoregional lymph nodes followed by the bones und ultimately visceral metastases [2]. Bones are the most common metastatic site in advanced PCa and associated with worse outcome than in patients with lymph node metastasis only [2].

Androgen deprivation therapy (ADT) is considered as standard treatment for metastatic disease [3]. When cancer cells no longer respond to ADT despite achieving castration levels of testosterone and prostate specific antigen (PSA) rises, PCa has become castration-resistant and is known as (metastatic) castration-resistant prostate cancer (mCRPC). Metastastic CRPC is the most advanced stage of PCa and is responsible for the vast majority of prostate cancer related deaths [4].

Patients with bone-metastatic CRPC (bmCRPC) are at higher risk of developing complications such as fractures according to their metastatic burden.

Patients with mCRPC can be treated with chemotherapy (Docetaxel, Cabazitaxel), next-generation androgen receptor-targeted agents (ARTAs) (Abiraterone or Enzalutamide), Sipuleucel-T, Radium-223, Olaparib or Rucaparib [5–14].

Enzalutamide is an oral 2nd generation androgen receptor (AR) antagonist which binds to the AR with higher relative affinity than for example the 1st generation AR antagonist bicalutamide. By suppressing nuclear translocation of the AR and its binding to coactivating proteins and DNA, Enzalutamide induces apoptosis [15].

Enzalutamide is approved for the treatment of asymptomatic or mildly symptomatic mCRPC patients in the pre-chemotherapy setting and after taxane treatment [13, 14]. It prolongs overall survival (OS) in both, mCRPC and in non-metastatic CRPC [13, 14, 16].

The determination whether therapy is efficacious is a challenging aspect for clinicians treating bmCRPC patients. Computer tomography (CT) imaging, magnetic resonance imaging (MRI) and bone scintigraphy can help to provide answers [17]. However, changes in the size of bone metastases are difficult to detect under early treatment. Bone-flare may occur representing detection of initially occult bone metastasis which become visible by increased activity of osteoblasts and hence osteosclerosis after response to treatment. This condition can be falsely interpreted as progressive disease (PD) [18]. In this setting, the clinical condition of the patient (e.g. Eastern Cooperative Oncology Group performance status (ECOG)) and the conventional biomarker PSA are commonly used to differentiate between treatment response and progress. Additionally, lactic acid dehydrogenase (LDH), alkaline phosphatase (ALP) and circulating tumor cells (CTCs) are under discussion to improve the selection of patients with superior benefit from Enzalutamide treatment [19–22].

PSA is widely used as screening marker for prostate cancer and for treatment monitoring in the setting of mCRPC. Data suggest that changes of PSA-values have prognostic potential and might help deciding whether to continue or stop therapy [23]. On the one hand, a PSA decline under therapy with Enzalutamide is associated with better OS, progression-free survival (PFS) and pain response [24, 25]. On the other hand, rising PSA-values alone are not a criterium for progression. Additionally, rising values under early treatment may occur before a delayed decline becomes evident. This phenomenon is called PSA-surge and characterized by rising PSA-levels after therapy initiation followed by a decline within the first 12 weeks of treatment [23, 26, 27]. Consequently, patients who do not show a PSA-decline ≥50% are difficult to evaluate. Therefore, the early differentiation between PSA-surge, potential bone-flare, and PD is important and further information is needed to distinguish between a true progression and a surge/flare.

Changes in CTC enumeration can prognosticate outcome of patients with mCRPC. Therefore, these changes are under discussion to support monitoring of treatment success [22]. Unfortunately, CTC-detection is not part of the clinical routine and not broadly available and therefore, probably not a suitable marker for repeated evaluation of response to therapy in routine medicine.

LDH and ALP have been shown to have prognostic potential as biomarkers. If found within normal range or if normalizing under therapy, they are associated with better survival in mCRPC-patients [19–21].

LDH is an unspecific biomarker. While rising LDH levels suggest poor prognosis, LDH-normalization suggests response to the therapy and indicates towards increased OS [28].

In contrast, ALP is more specific in bmCRPC and can provide prognostic information [20, 21, 29].

Furthermore, a phenomenon called ALP-bouncing, defined as a rising ALP during the first 2–8 weeks after starting therapy and followed by a decline to baseline levels or below showed to be associated with response and outcome in bmCRPC treated with Abiraterone [20]. Other data confirmed this finding; hence, ALP could be a promising biomarker during the first weeks of therapy helping to decide whether to continue or stop therapy early [20].

For LDH-normalization, ALP-bouncing, and PSA-decline as well as the combination of these biomarkers it was shown that these may help identifying patients with good response to therapy with Enzalutamide [30]. According to these results, showing that changes of LDH and ALP add information in patients with or without significant PSA-decline, we intended to study the prognostic potential of these markers in patients with questionable response to therapy because of an insignificant PSA-decline.

Therefore, we studied patients receiving Enzalutamide without initial PSA-response and evaluated LDH and ALP levels as potential prognostic factors of survival outcomes during the remaining study period.

## Methods

### Patient population and outcome evaluation

 We retrospectively reviewed 99 mCRPC patients who received Enzalutamide at the Department of Urology of the University Hospital Münster, Germany between 07/2010 and 09/2019.

 Prior to any study related activity, the patients had given written informed consent before participating and the ethics committee-approval was granted. (Aktenzeichen: 2007-467-f-S) The study was carried out according to the requirements of the declaration of Helsinki.

Since ALP is irrelevant when bone metastases are missing, we excluded patients with non-bone-metastatic disease. Further, we excluded the patients with a significant PSA-decline (≥50%) to determine the benefit of changes of ALP and LDH when PSA is leading to non-straightforward information. Finally, 48 patients with a complete data set were evaluable for analysis.

All patients received Enzalutamide according to the approved label in a pre-chemotherapy setting (*n* = 21 (43.8%)) or after docetaxel chemotherapy setting (*n* = 27 (56.3%). Twelve patients (25.0%) received Enzalutamide in a pre-Abiraterone setting. Thirty-four patients (70.8%) were on a stable dose of a bone targeting agent (zoledronic acid *n* = 17 (35.4%) or denosumab *n* = 17 (35.4%)) for at least three months before initiation of Enzalutamide treatment. The other men did not receive bone health agents at all.

Directly prior to the start of Enzalutamide, blood was drawn for baseline analysis. In addition, we evaluated ECOG and pain level. The follow-up examinations were performed after two and four weeks and every 4 weeks thereafter. PSA, ALP and LDH levels in serum samples were immediately measured on the same day.

Patients were grouped as with normal LDH (all values in the range of normal during the whole study period) or patients with elevated LDH levels prior to start of therapy with Enzalutamide and conversed to normal levels and stayed there during the remaining study period.

ALP-bouncing was previously defined as a rising ALP during the first 2–8 weeks of therapy followed by a decline to or below pre-treatment levels. Rising ALP was defined as any increase during the first 12 weeks of Enzalutamide.

The assessment of current response status took place at the routinely planned visits. For the determination of response status, ECOG, presence of pain, laboratory constellations as well as imaging were taken into account. Clinical progression was defined as symptomatic progression (worsening or new prostate cancer-related symptoms). PSA progression was defined according to the ‘Prostate Cancer Working Group 3 (PCWG3) criteria’ as a confirmed increase of 25% or greater and a value of more than 2 ng/ml from baseline beyond 12 weeks [17]. When no clinically and biochemically progression was suspected, imaging was not performed routinely. When progression was asumed, soft tissue metastases were evaluated by CT- and/or MRI-scans of thorax, abdomen and pelvis. Bone metastases were assessed by bone scans. In 36 patients imaging was performed and PD was defined according to Response Evaluation Criteria in Solid Tumours (RECIST) 1.1 criteria for cross-sectional imaging and by PCWG3 criteria for bone scans [17, 31].

## Statistical methods

We used SPSS statistics V.26 (IBM Inc., Armonk, NY) for statistical assessment.

The descriptive statistics are reported as medians with interquartile ranges (IQR) or 95% confidence intervals (CI) for continuous variables and as frequencies and populations for categorical variables.

Regarding the differences between ALP-increase vs. no increase, ALP-bouncing vs. no bouncing and LDH-normalization vs. no normalization as well as the combination of these biomarker changes, survival analyses were performed using Kaplan-Meier-Analyses (KMA). The definition of PFS included biochemical and radiographic progression according to the definition of PCWG3 and RECIST 1.1. OS was defined as the interval from treatment initiation until death from any cause.

For univariate (UV) and multivariate (MV) analyses of the significance of survival outcomes for the different biomarkers we used Cox regression models.

Hazard ratios (HR) are given with 95% CI. All reported p-values are two-sided and statistical significance was assumed with a P < 0.05.

Considering OS, statistical power was estimated 13.36% for LDH-normalization, 41.18% for rising ALP at 12 weeks and 49.7% for rising ALP at 12 weeks without LDH-normalization. For the analysis of PFS it was 12.43%, 33.12% and 29.21%, respectively.

## Results

### Characteristic of the study group

Descriptive characteristics of the cohort are presented in Table 1. The median age of our patients was 70.5 years (IQR, 63.0-75.8 years). At start of Enzalutamide, lymphonodal metastases were present in 38 patients (79.2%) and visceral metastases in 13 patients (27.1%). A Gleason-Score of ≥ 8 at initial diagnosis was present in 23 patients (47.9%). Considering ECOG performance status, 24 (50.0%) of our patients were asymptomatic and fully active (ECOG grade 0) while 19 patients (39.6%) were ECOG grade 1, 4 (8.3%) ECOG grade 2, and 1 (2.1%) ECOG grade 3, respectively. The proportions of patients in either pre- or post-chemotherapy setting showing rising ALP at 12 weeks, LDH-normalization, or ALP rising at 12 weeks, without LDH-normalization are given in Table 1.

The median follow-up was 12 months (IQR, 7.0-19.3 months). The median time on Enzalutamide was 5 months (IQR, 3.0–9.0 months). Median baseline levels were 125.3 ng/ml (IQR, 51.2-470.8) for PSA, 274.0 U/l (IQR, 232.0-352.0) for LDH and 155.0 U/l (IQR, 97.8-304.3) for ALP. A PSA-surge occurred in 10 patients (20.8%). Only three of them (30%) had a PD, seven patients (70%) subsequently responded to Enzalutamide. Three patients (6.3%) showed to have an ALP-bouncing (Table 1).

**Table 1:** Characteristics of patients with bmCRPC on Enzalutamide without PSA-response (decline ≥ 50%)

Variable	all
**Patients [n], (%)**	48 (100)
**Age, median [years] (IQR)**	70.5 (63.0-75.8)
**Bone metastases [n] (%)**	48 (100)
**Lymph node metastases [n] (%)**	38 (79.2)
**Visceral metastases [n] (%)**	13 (27.1)
**Pre chemotherapy [n] (%)**	21 (43.8)
**Post chemotherapy [n] (%)**	27 (56.3)
**Enzalutamide pre Abiraterone [n] (%)**	12 (25)
**Antiresorptive therapy [n] (%)** **Zoledronic acid [n] (%)** **Denosumab [n] (%)**	34 (70.8) 17 (35.4) 17 (35.4)
**ECOG (all) [n] (%)** **0** **1** **2** **3**	24 (50.0) 19 (39.6) 4 (8.3) 1 (2.1)
**Gleason-Score ≥ 8 [n] (%)**	23 (47.9)
**Median ALP at baseline [U/l] (IQR)**	155.0 (97.8-304.3)
**Median LDH at baseline [U/l] (IQR)**	274.0 (232.0-352.0)
**Median PSA at baseline [ng/ml] (IQR)**	125.3 (51.2-470.8)
**LDH at baseline > UNL [n] (%)**	39 (81.3)
**Median duration of therapy with Enzalutamide [months] (IQR)**	5.0 (3.0–9.0)
**Median follow up [months] (IQR)**	12.0 (7.0-19.3)
**Patients died during follow up [n] (%)**	41 (85.4)
**Best clinical outcome [n] (%)** **Complete remission** **Partial remission** **Stable disease** **Progressive disease**	0 (0) 3 (6.3) 30 (62.5) 15 (31.3)
**PD vs. all [n] (%)**	15 (31.3)
**PSA-surge [n] (%)**	10 (20.8)
**LDH-normalization [n] (%)** **Enzalutamide pre chemotherapy [n] (%)** **Enzalutamide post chemotherapy [n] (%)**	8 (16.7) 3 (6.3) 5 (10.4)
**ALP rising at 12 weeks [n] (%)** **Enzalutamide pre chemotherapy [n] (%)** **Enzalutamide post chemotherapy [n] (%)**	21 (43.8) 8 (16.7) 13 (27.1)
**ALP rising at 12 weeks, without LDH normalization [n] (%)** **Enzalutamide pre chemotherapy [n] (%)** **Enzalutamide post chemotherapy [n] (%)**	18 (37.5) 7 (14.6) 11(22.9)
**ALP-bouncing [n] (%)**	3 (6.3)
**Number of following therapies after Enzalutamide [n] (%)** **0** **1** **2** **3** **4**	15 (31.3) 15 (31.3) 14 (29.2) 3 (6.3) 1 (2.1)
Abbreviations: bmCRPC: bone metastatic castration-resistant prostate cancer; ALP: alkaline phosphatase; LDH: lactate dehydrogenase; PSA: prostate specific antigen; UNL: upper normal limit; ECOG: eastern co-operative oncology group; CR: complete remission; PR: partial remission; SD: stable disease; PD: progressive disease, IQR: interquartile range	

## Kaplan-Meier survival analysis

**Fig. 1:**
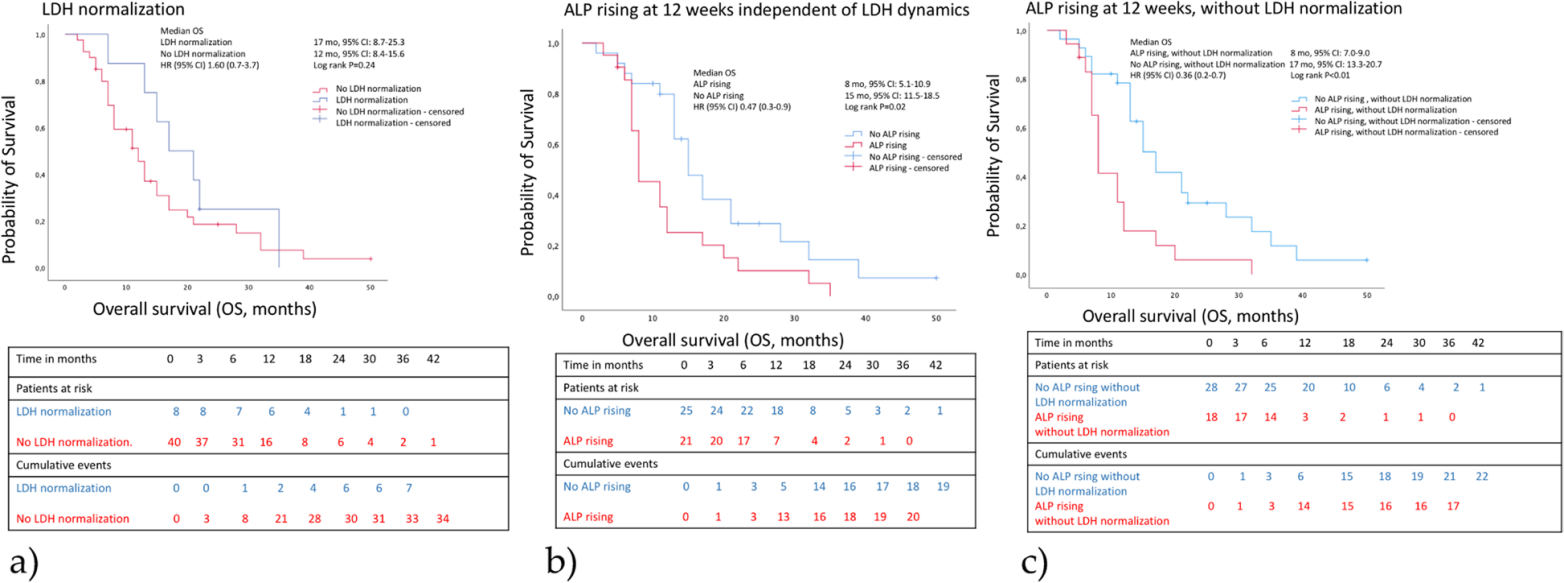
Kaplan-Meier analyses of overall survival of mCRPC patients treated with Enzalutamide who do not show a sufficientent initial PSA decline ≥ 50% **a**) with and without LDH normalization **b**) with and without ALP rising at 12 weeks independent of LDH dynamics, and **c**) with and without ALP rising at 12 weeks, without LDH normalizastion

**Fig. 2:**
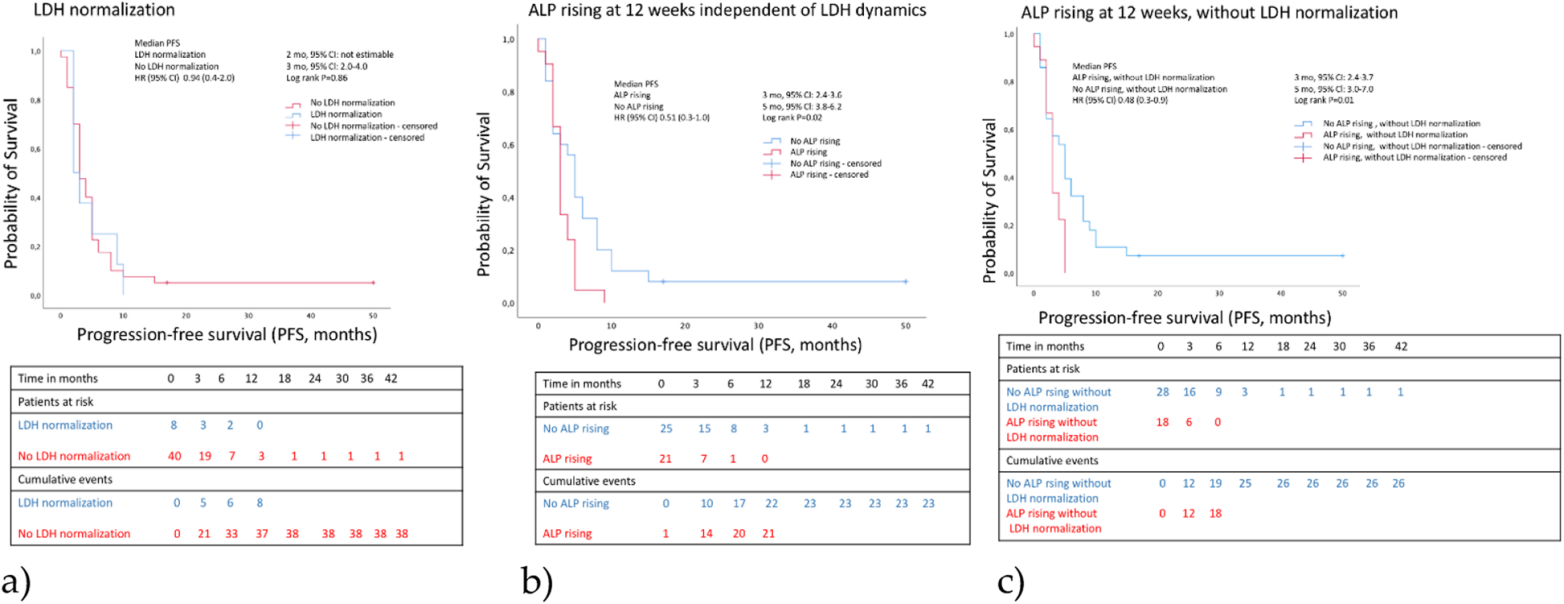
Kaplan-Meier analyses of progression free survival of mCRPC patients treated with Enzalutamide who do not show a sufficientent initial PSA decline ≥ 50% **a**) with and without LDH normalization **b**) with and without ALP rising at 12 weeks independent of LDH dynamics, and **c**) with and without ALP rising at 12 weeks, without LDH normalizastion

The Kaplan-Meier analyses for PFS and OS are given in Figs. 1 and 2. Considering survival of the overall population, median PFS resulted in 3 months (95%CI, 2.0–4.0 months), median OS in 13 months (95%CI, 10.9–15.1 months). Regarding subgroups, PFS and OS worsen with rising ALP at 12 weeks alone and in combination of rising ALP at 12 weeks without LDH-normalization. The changes are associated with a shorter PFS of 3 months (95%CI, 2.4–3.6) vs. 5 months (95%CI, 3.8–6.2) (Log rank *P* = 0.02) and 3 months (95%CI, 2.4–3.7) vs. 5 months (95%CI, 3.0–7.0) (*P* = 0.01), respectively.

The analysis of OS showed similar results with 8 months (95%CI, 5.1–10.9) vs. 15 months (95%CI, 11.5–18.5) (*P* = 0.02) for ALP rising at 12 weeks, 8 months (95%CI, 7.0–9.0) vs. 17 months (13.3–20.7) (*P* < 0.01) for ALP rising at 12 weeks without LDH-normalization.

LDH-normalization did not predict PFS with 2 months (95%CI, not estimable) vs. 3 months (95%CI, 2.0–4.0) (*P* = 0.86) and the OS with 17 months (95%CI, 8.7–25.3) vs. 12 months (95%CI, 8.4–15.6) (*P* = 0.24).

### Changes of LDH and ALP as prognostic markers

In univariate (UV) analysis, parameters with unfavorable changes were associated with a worse outcome. These results are displayed in Table 2. In the analysis of PFS, ALP rising at 12 weeks alone, ALP rising at 12 weeks without LDH-normalization and the application of Enzalutamide after chemotherapy showed a statistically significant association towards shorter PFS (HR: 0.51 (95%CI, 0.3-1.0); *P* = 0.04; HR: 0.48 (95%CI, 0.3–0.9); *P* = 0.03; HR: 0.48 (95%CI, 0.3–0.9); *P* = 0.03).

Worse OS was significantly associated with ALP rising at 12 weeks alone, ALP rising at 12 weeks without LDH-normalization, and the application of Enzalutamide after chemotherapy (HR: 0.47 (95%CI, 0.3–0.9); *P* = 0.02; HR: 0.36 (95%CI, 0.2–0.7); *P* < 0.01; HR: 0.31 (95%CI, 0.2–0.7); *P* < 0.01).

**Table 2:** Univariate analyses of significant biomarkers for PFS and OS in 48 bmCRPC-patients on Enzalutamide-therapy without PSA-response

Progression Free Survival			Overall Survival		
**Variable**	**HR (95% CI)**	**P**	**Variable**	**HR (95% CI)**	**P**
**Visceral metastases** **Yes** **No**	1 (reference) 0.71 (0.37–1.36)	0.30	**Visceral metastases** **Yes** **No**	1 (reference) 0.95 (0.46–1.94)	0.88
**Enzalutamide after chemotherapy** **Yes** **No**	1 (reference) 0.48 (0.25–0.92)	**0.03**	**Enzalutamide after chemotherapy** **Yes** **No**	1 (reference) 0.31 (0.15–0.65)	**< 0.01**
**Gleason-Score ≥ 8** **No** **Yes**	1 (reference) 1.22 (0.62–2.37)	0.57	**Gleason-Score ≥ 8** **No** **Yes**	1 (reference) 1.27 (0.62–2.58)	0.52
**LDH normalization** **Yes** **No**	1 (reference) 0.94 (0.44–2.03)	0.88	**LDH normalization** **Yes** **No**	1 (reference) 1.60 (0.71–3.65)	0.26
**ALP rising at 12 weeks** **Yes** **No**	1 (reference) 0.51 (0.27–0.98)	**0.04**	**ALP rising at 12 weeks** **Yes** **No**	1 (reference) 0.47 (0.25–0.91)	**0.02**
**ALP rising at 12 weeks, without LDH normalization** **Yes** **No**	1 (reference) 0.48 (0.25–0.94)	**0.03**	**ALP rising at 12 weeks, without LDH normalization** **Yes** **No**	1 (reference) 0.36 (0.19–0.71)	**< 0.01**
**ECOG > 0** **Yes** **No**	1 (reference) 0.67 (0.36–1.23)	0.19	**ECOG > 0** **Yes** **No**	1 (reference) 0.71 (0.38–1.32)	0.28
**Age > 70 years** **Yes** **No**	1 (reference) 0.66 (0.36–1.18)	0.16	**Age > 70 years** **Yes** **No**	1 (reference) 0.73 (0.4–1.37)	0.33
Abbreviations: PFS: progression-free survival; OS: overall survival; HR: hazard ratio; 95% CI: 95% confidence interval; ALP: alkaline phosphatase; LDH: lactate dehydrogenase; bmCRPC: bone metastatic castration-resistant prostate cancer; PSA: prostate specific antigen, ECOG: eastern cooperative oncology group performance status					

The results displayed in Table 3 show that in multivariate (MV) analysis, regarding PFS, none of the parameters remained an independent prognostic factor for worse PFS. Within the analysis of OS, only the application of Enzalutamide after chemotherapy showed an independent and statistically relevant difference towards shorter OS (HR 0.36 (95%CI, 0.2–0.8); *P* = 0.01) .

**Table 3:** Multivariate analyses of biomarkers for PFS and OS in 48 bmCRPC-patients on Enzalutamide-therapy without PSA-response

Progression Free Survival			Overall Survival		
**Variable**	**HR (95% CI)**	**P**	**Variable**	**HR (95% CI)**	**P**
**ALP rising at 12 weeks** **Yes** **No**	1 (reference) 0.77 (0.22–2.69)	0.68	**ALP rising at 12 weeks** **Yes** **No**	1 (reference) 1.14 (0.32–4.09)	0.83
**ALP rising at 12 weeks, without LDH normalization** **Yes** **No**	1 (reference) 0.70 (0.18–2.67)	0.6	**ALP rising at 12 weeks, without LDH normalization** **Yes** **No**	1 (reference) 0.31 (0.08–1.13)	0.08
**Enzalutamide after chemotherapy** **Yes** **No**	1 (reference) 0.55 (0.28–1.09)	0.09	**Enzalutamide after chemotherapy** **Yes** **No**	1 (reference) 0.36 (0.17–0.78)	**0.01**
**ECOG > 0** **Yes** **No**	1 (reference) 0.78 (0.41–1.49)	0.44	**ECOG > 0** **Yes** **No**	1 (reference) 0.77 (0.39–1.52)	0.45
**Visceral metastases** **Yes** **No**	1 (reference) 0.92 (0.44–1.92)	0.82	**Visceral metastases** **Yes** **No**	1 (reference) 0.95 (0.44–2.05)	0.89
Abbreviations: PFS: progression-free survival; OS: overall survival; HR: hazard ratio; 95% CI: 95% confidence interval; ALP: alkaline phosphatase; LDH: lactate dehydrogenase; bmCRPC: bone metastatic castration-resistant prostate cancer; PSA: prostate specific antigen; ECOG: eastern cooperative oncology group performance status					

## Discussion

There are several clinical and biochemical prognostic factors that can be captured prior to treatment or during very early treatment that are associated with survival outcomes. PSA, PSA kinetics, LDH, ALP, hemoglobin, performance status, presence of metastases, presence of pain, CTCs, Gleason Score, age, and albumin have been under discussion to be prognostic in mCRPC [32–34].

However, there are some limitations considering these factors. Clinical factors (ECOG and pain level) are difficult to compare since the determination of those markers is highly dependent on both the subjective reporting of the patient and the evaluator. Further, many patients are only mildly symptomatic or even asymptomatic, thus, a change of symptoms to the better cannot occur in these patients. Hemoglobin, age, and LDH and albumin are not specific for prostate cancer.

Specific biomarkers which are easily available and might help to prognosticate treatment outcomes for patients under early therapy with Enzalutamide are therefore essential but currently lacking [30].

CTCs are specific and an increase under therapy with Enzalutamide was shown to be associated with worse PFS and OS. Additionally, a prospective, multicenter study showed that CTC enumeration is an independent prognostic factor [35, 36]. Another study showed that CTC dynamics are more prognostic than post-therapy changes in PSA [36]. But CTC-assays are expensive and not easily available. This makes the use in clinical routine difficult.

To optimize treatment, easily available and affordable biomarkers like PSA, LDH and ALP would be better options in wide clinical routine.

PSA is already widely used as a biomarker in prostate cancer, in mCRPC particularly, for treatment monitoring. A decrease after therapy initiation can be interpreted as a surrogate for expected response. Especially a decrease of PSA-levels by ≥50% is regarded as threshold of biochemical response to a given treatment [17]. Despite the given fact, that declining PSA-values in most cases indicate response to therapy, a transient increase of PSA may occur not due to a true progression but by circulatory release of PSA during response of a newly started therapy, as well. Furthermore, some patients only show stabilization of PSA-values and do not show a progression either [37]. Hence, the group of patients that shows a PSA-decline < 50% is more difficult to evaluate and might even be misinterpreted as therapeutic failure [38]. A PSA-surge can be regularly observed [23, 26, 27]. This clinically difficult situation is especially demanding in mildly- or asymptomatic patients, when PSA is the only clinical measure available.

For bone metastatic disease, a phenomenon in imaging, comparable to an initial PSA-increase, a bone-flare, is known. This can occur when imaging is performed during the first three to six months of therapy and shows a pseudoprogression by visualized hitherto occult metastases and represents response to treatment but is often time mistaken for pressive disease [18, 39–41].

In our study 10 patients (20,8%) showed a PSA-surge with rising PSA-levels after therapy initiation that started to decline after 12 weeks of treatment. Out of these patients, only three (30%) ultimately showed a progressive disease at any time of evaluation. This finding underlines guideline recommendations saying that changes in PSA under treatment should not be used alone when deciding whether to continue treatment [17].

In the worst scenario a misinterpreted rising PSA could lead to either premature termination of a working treatment or on the other hand to a shift to the next line in therapy when it is too late and a patient is no more eligible to receive for example chemotherapy. Considering our patients, seven (70%) ultimately responded to Enzalutamide and would have been imperiled by terminating treatment too early in case of overestimating the value of PSA or in the case of a lack of other criteria. We tried to find a biomarker to differentiate between patients with a PSA-surge and those with a true progression by separating these 10 patients into two groups. Probably due to a small group size of only three patients with progressive disease, statistical analysis did not result in meaningful differences. Though a number of only 10 patients with PSA-surge was not sufficient for statistical analysis, our result emphasizes the statement that we need other biomarkers, alone or in addition to accepted ones, which help to distinguish between response and progression in the given scenario. Therefore, we evaluated additional and easily available biomarkers for prognostication of outcome in this highly selected population without significant PSA-decline (< 50%).

LDH is a very unspecific biomarker for malignancies in general and for inflammatory diseases [42]. Nevertheless, rising LDH levels are associated with worse prognosis, whereas normalization can be considered a marker of response to therapy and better OS in cancer patients [28].

ALP is more specific, and bone related and can provide prognostic information for men with PCa [20, 21, 29]. In a metaanalysis of unselected PCa cohorts, elevated ALP was associated with worse PFS and OS [43]. According to other data, patients in the setting of newly diagnosed prostate cancer, can be divided in three different risk groups, low corresponds to zero risk factors, intermediate to one or to two risk factors and high corresponds with three risk factors depending on the changes of ALP, PSA and hemoglobin (Hb) under therapy predicting PFS and OS. [44].

In recent studies, ALP-bouncing was found to be prognostic for better survival outcomes. It was defined as a rising ALP during the first 2–8 weeks after starting therapy and followed by a decline to or below pre-treatment and baseline levels. The authors concluded that it might be a promising prognostic biomarker in patients with bmCRPC [20].

Further studies suggested that LDH-normalization, ALP-bouncing, PSA-decline, and the combination of these three biomarkers could help identifying patients with a good response to therapy with Enzalutamide [30]. This study evaluated patients with bmCRPC with or without significant PSA-decline.

The purpose of this study was to evaluate the prognostic ability of dynamic changes of ALP and LDH levels as a prognostic factor of PFS and OS in patients with bmCRPC that do not show a sufficient PSA-decline of ≥50% of the initial value in the early therapy with Enzalutamide. In our cohort, we evaluated several dynamic biomarker changes. Considering LDH-normalization or ALP-bouncing alone we were not able to show that these, usually regarded as favorable, can be considered as prognostic factors for better PFS or OS.

LDH-normalization but also a decline under therapy with Enzalutamide cannot be used as a predictor of treatment success for our cohort. The Kaplan-Meier analysis did not reveal significantly better PFS or OS. The non-specific nature of LDH in the bone-metastases enriched population in our study may be part of the reasons for this finding.

With respect to the very small number of only three patients with ALP-bouncing (6.3%) in our study, we concluded that the phenomenon is very rare, at least in our selected cohort with only patients without significant PSA-decline. Therefore, we could not evaluate this criterion.

However, we found in UV that sole ALP rising at 12 weeks and ALP rising at 12 weeks without LDH-normalization under therapy with Enzalutamide are prognostic factors of poor PFS and OS in patients with bmCRPC. Still, in our view, the favorable results considering the combination of rising ALP without LDH-normalization, should not be overinterpreted. On the one hand, we could only show statistical relevance in UV whereas MV did not confirm these results and, on the other hand, probably, ALP accounts for most of this effect. Hence, ALP could be a useful biomarker to differentiate between PSA-surge and PSA-progression in bmCRPC patients during the treatment with Enzalutamide.

Translating our results into clinical routine and looking at the extreme outliners, there might be a group of patients benefiting from a change of therapy. Six of the 21 patients with rising ALP-values showed extreme changes which, from a retrospective view, could have benefited from treatment adjustment. Those patients had ALP-values that, after 12 weeks, doubled at least. Survival was were extremely short in this group, PFS was 2.5 (2-4.25) and OS 6.5 (4.75-8) months. In the context of the whole study cohort, PFS was 3 months for patients with rising ALP after 12 weeks of treatment, OS was 8 months for this group. Patient without rising ALP had a PFS of 5 and an OS of 17 months.

 Our study is limited due to its relatively small cohort of only 48 patients recruited in a single center and by problems inherent to the retrospective approach, e.g. a missing validation cohort. We separated these patients into subgroups, by which the group sizes became even smaller. This is probably one reason why we were not able to demonstrate significant results. Therefore, larger prospective trials are needed to validate the significance of our findings.

Nonetheless, our results are clinically important. In clinical practice it is difficult to differentiate between a continuous rise, that would represent progression, and a PSA-surge which is followed by a response or stable disease. ALP is easily accessible and can help us to make the right decision in individual cases.

## Conclusions

Dynamic changes of ALP and LDH (non-rise and normalization) under therapy with Enzalutamide may be associated with clinical benefit and better PFS and OS in patients with bmCRPC who do not show a significant PSA decline. Potentially ALP is the more relevant parameter since it is specific for bone metastatic disease and LDH-normalization alone could not show a trend towards improved PFS or OS.

## Data Availability

Analyzed data are stored at the Department of Urology, University hospital Münster, Germany and are available on reasonable request if not already included in this article.

## Abbreviations

PCa: Prostate cancer
mCRPC: Metastatic castration resistant prostate cancer
bmCRPC: Bone metastatic castration resistant prostate cancer
ADT: Androgen-deprivation therapy
LDH: Lactic acid dehydrogenase
PSA: Prostate specific antigen
ALP: Alkaline phosphatase
AR: Androgen receptor
ECOG: Eastern cooperative oncology group performance status
CTC: Circulating tumor cells
PD: Progressive disease
OS: Overall survival
PFS: Progression-free survival
CT: Computer tomography
MRI: Magnetic resonance imaging
IQR: Interquartile range
PCWG 3: Prostate Cancer Working Group 3
RECIST: Response Evaluation Criteria in Solid Tumours
CI: Confidence interval
KMA: Kaplan Meier analysis
UV: Univariate
MV: Multivariate
HR: Hazard ratio
Hb: Hemoglobin
ARTAs: Androgen receptor-targeted agents

## Declarations

** Consent for publication**

Author KS has received honoraria for advisory-roles from Amgen, AstraZeneca, Bayer, Janssen Cilag and Ilpsen.
